# Syphilitic Chlorosis

**Published:** 1845-02

**Authors:** 


*Syphilitic Chlorosis,* by M. Ricord.—In a great number of researches that M. Ricord has made upon the blood of persons affected with syphilis, he has constantly found the number of globules diminished in variable proportions. This state of the blood is what M. Ricord calls syphilitic chlorosis; and it has indeed many points of resemblance to other kinds of chlorosis. It is to this affection that we are to attribute the particular color that the skin of patients affected with constitutional syphilis presents; as in chlorosis, the physical and moral debility indicates a trouble of the circulation. The discolored skin, the inanimate expression of the eyes, shows the blood no longer possesses its normal qualities. Syphilitic chlorosis, generally, exists before any secondary or tertiary symptom has declared itself. Its principal characters are, beside the general aspect that we have just mentioned, considerable prostration, with pains in the neighborhood of the articulations, having nocturnal exacerbations, unacompanied, however, by swelling or change of color, and which are not augmented by pressure. Cephalagia, neuralgia of the fifth pair, paralysis of the facial nerve, are common signs. Alopecia, engorgement of the of the posterior or lateral cervical ganglions and sometimes only the mastoid ganglions, complete this group of phenomena, which is rarely preceded or accompanied by febrile movement.

This particular state of the blood grows worse as the syphilitic infection gives place to secondary or tertiary symptoms. It may continue in different degrees after they disappear. The first conclusion to be drawn from these considerations is, that syphilis being an anemic disease, or, at least, always complicated with anemia, the antiphlogistic method of treatment is dangerous. The second conclusion is, the necessity of a nutritious diet. "The treatment that I adopt," says M. Ricord, "consists in the combination of ferruginous and mercurial preparations, if there exist no counter indications. When the secondary symptoms pass to tertiary, the mercurials, combined with iodide of iron, or with iodide of potassium, suffice to reconstitute the blood.—*Bulletin Generale Therapeutique.*

